# Electrowetting on Dielectric (EWOD) Based Portable Multimaterial Printer To Fabricate Origami Devices

**DOI:** 10.1021/acsami.5c12629

**Published:** 2025-07-31

**Authors:** Yuhi Watanabe, Atsushi Matsushita, Mutsuki Matsumoto, Yusuke Akitsu, Yu Kuwajima, Hiroki Shigemune

**Affiliations:** † Electrical Engineering and Computer Science, Graduate School of Engineering and Science, 47745 Shibaura Institute of Technology, 3-7-5,Toyosu, Koto-ku, Tokyo 135-8548, Japan; ‡ Department of Mechanics, Mathematics and Management (DMMM), 18951 Politecnico di Bari, Via Orabona 4, Bari 70125, Italy; § College of Engineering, 47745 Shibaura Institute of Technology, 3-7-5,Toyosu, Koto-ku, Tokyo 135-8548, Japan

**Keywords:** soft robotics, multimaterial printer, electrowetting, self-folding, origami devices, portable

## Abstract

Origami devices are expected to be applied in fields such as space exploration, medicine, and agriculture and are being extensively researched in both scientific and engineering contexts. However, the difficulty of fabrication is high, and it is particularly challenging to fabricate them on-demand and on-site with a compact device. We have a technology for automatically fabricating origami devices by printing conductive and insulating solutions on paper. In this study, we have developed a portable, multimaterial printer using electrowetting on dielectric (EWOD) technique that drives both conductive and insulating liquids. We overcame the low portability of conventional inkjet printers and achieved a palm-sized compact printer. Specifically, we used EWOD to promote the driving of liquid within the channels printed on paper and investigated the electrical input, channel, and electrode designs necessary for proper control. We successfully drove both insulating and conductive liquids and evaluated the printing performance and precision. As a demonstration, we successfully fabricated an origami stretchable strain sensor and a breath sensor using the proposed system and verified the durability of the origami device through repeated testing. The development of a portable control circuit that generates the investigated electrical input signals has enabled the rapid and convenient fabrication of 3D devices without location constraints, potentially accelerating the adoption of IoT devices.

## Graphical abstract

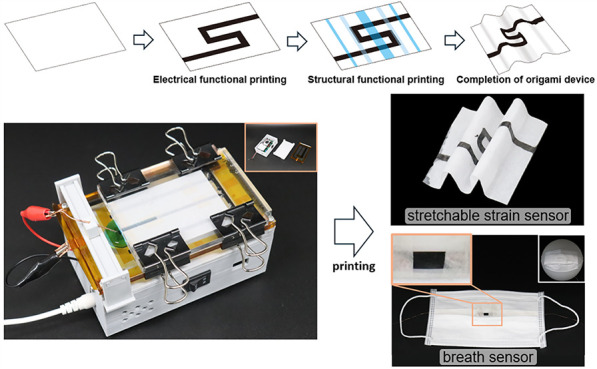

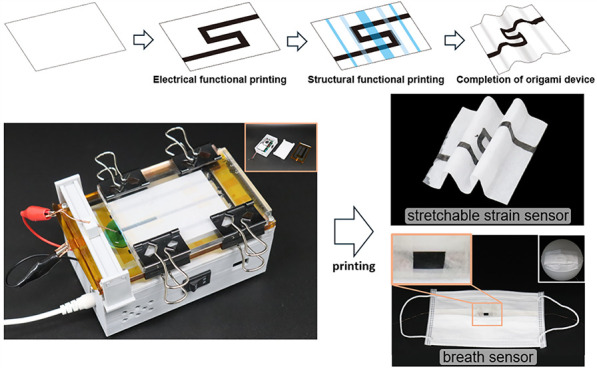

## Introduction

In the current fourth industrial revolution, there is a growing demand for multivariety and small-lot production and tailor-made capabilities. This paradigm shift is also emphasized in the outlook of the fifth industrial revolution, where a transition to a sustainable industrial structure, equipped with adaptability to environmental changes and centered on human needs, is required. The ability to fabricate rapidly and conveniently not only enables human-centered research and development through repeated trial and error, however also provides the advantage of preparing required functionalities on-demand in locations such as factories, medical facilities, farms, and space.^1^ Although it is possible to quickly fabricate 3D structures, rapid prototyping of 3D devices remains challenging. Since 3D electronic devices have electronics embedded within their structure, they become lightweight and compact, which makes them promising for applications such as wearable devices,^2^ automotive electronics,^3^ and robotic systems for extreme environment exploration.^4^ However, as this represents an entirely new method of device fabrication, further exploration of potential applications is anticipated.

There are three main approaches proposed for fabricating 3D devices. The first approach utilizes a multimaterial 3D printer.^5,6^ Micalizzi et al. developed a shape memory actuator using a multimaterial 3D printer capable of simultaneously printing conductive and insulating inks.^7^ It is possible to directly embed electronics within structures formed by a 3D printer. The second approach involves directly drawing conductive ink on a preprepared structure using 3D Computer Numerical Control (CNC) technology.^8^ While it is possible to draw wiring on the surface of the structure, it is challenging to draw wiring within the structure or in hidden areas. The third approach involves folding 2D circuits using origami techniques.^9^ Conventional pick-and-place and lithography techniques can be applied to fabricate 2D circuits, which provides the advantage of transforming high-resolution circuits into 3D configurations.

We had proposed paper mechatronics approach to realize 3D origami devices by forming electronics on paper and enabling self-folding.^10^ By using a single inkjet printer, we print both circuit-forming and structure-forming inks to produce the 3D origami device through self-folding of the paper with the printed circuit.^11^ Self-folding automatically generates the 3D origami structures by driving the material itself.^12–14^ We can develop origami devices and robots by combining conductive materials with self-folding sheets.^15–17^ Origami devices are attracting attention due to their ability to imbue electronics with stretchability, impact absorption, and deployability simply through folding.^18–20^

Our method for fabricating origami devices offers the advantage of using a single inkjet printer. However, current inkjet printers face the challenge of low portability, which renders them impractical for use in large agricultural fields, controlled medical environments, or space missions where space is at a premium and versatility is crucial. To overcome these limitations, we develop a compact multimaterial printer using electrowetting on dielectric (EWOD) technology in this paper, with the aim of expanding the application scope of paper mechatronics. While other electrically driven fluid control methods, such as electro hydro dynamics (EHD)^21,22^ and magneto hydro dynamics (MHD),^23,24^ exist, each has limitations: EHD can only drive insulating liquids, whereas MHD is restricted to conductive liquids. EWOD, in contrast, can drive both conductive and insulating liquids and is thus ideal for a versatile multimaterial printer.

Conventional printing techniques such as stencil printing and screen printing offer high throughput and simple mechanical structures, making them well suited for mass production. In contrast, the EWOD-based printing system developed in this study exhibits distinct advantages in terms of miniaturization, portability, and controllability. Specifically, liquid manipulation in our system is achieved solely through electrical ON/OFF signals, eliminating the need for mechanical actuators. This structural simplification has enabled the realization of a palm-sized portable system, and we have successfully constructed a prototype with potential integration into mobile platforms. Furthermore, our approach allows for dynamic switching between multiple types of inks, offering high flexibility in material selection and pattern design. These features are particularly advantageous not for large-scale manufacturing, but rather for on-site fabrication of deployable sensors and local prototyping. Based on these comparisons, we believe that the proposed system offers significant advantages in application domains distinct from those addressed by conventional printing technologies.

The proposed EWOD-based printer system enables printing on both the front and back surfaces of the substrate, which can later be reconstructed into a three-dimensional origami structure. By folding the prepatterned surfaces inward, electrodes and circuits can be embedded in locations that would otherwise be inaccessible after assembly. In this way, our approach fundamentally differs from conventional direct writing methods by allowing circuit layout to be integrated into the structural design. This provides a new degree of freedom in constructing complex three-dimensional devices.

This paper proposes a multimaterial printer system utilizing enhanced capillary force through EWOD. This system electrically controls the inflow of ink into microchannels to print patterns onto paper. Printing the conductive solution forms electrodes on the paper, and the structure formation solution induces self-folding to create a 3D origami structure. The appropriate voltage waveform, frequency, and channel design for each solution were investigated to determine the conditions for precise and rapid solution inflow. Additionally, we examined the electrical and structural properties of the printed device, which demonstrates the capability of the printer to produce functional origami devices. As applications, we fabricated a stretchable strain sensor and a breath sensor and evaluated their performance to verify the practical applicability of the origami devices. Furthermore, we developed a compact high-voltage control circuit compatible with EWOD and integrated it into the printer system to achieve a palm-sized multimaterial printer.

We propose a method to regulate liquid inflow by electrically modulating the contact angle through electrowetting-on-dielectric (EWOD), in combination with fluidic resistance defined by channel geometry. This integrated mechanism enables voltage-controlled inflow into microchannels without the need for mechanical pumps or valves. To the best of our knowledge, such a strategy based on the interplay between EWOD actuation and fluidic resistance has not been proposed in prior studies across related fields. This concept forms a novel and foundational approach to multimaterial printing, and it also holds potential for broader applications in lab-on-a-chip platforms and paper-based IoT devices, where precise liquid handling is essential for reagent delivery, reaction control, and sensing. The multimaterial printer, capable of rapidly fabricating 3D devices on demand, contributes to the realization of Society 5.0 through IoT and enhances the applicability of paper-based devices.

## Results and Discussion

### EWOD Printing System

Figure 1a shows an overview of the EWOD printing system. With the paper clamped in the system, a solution is dropped onto it, and the printing pattern is formed by controlling the solution with the voltage applied between the solution and the electrode (Video S1). Figure 1b shows the fabrication process for the origami device. First, electrical functionality is imparted to the paper by printing a conductive solution. After that, the paper undergoes self-folding by printing the structure formation solution, completing the origami device.

**Figure 1:**
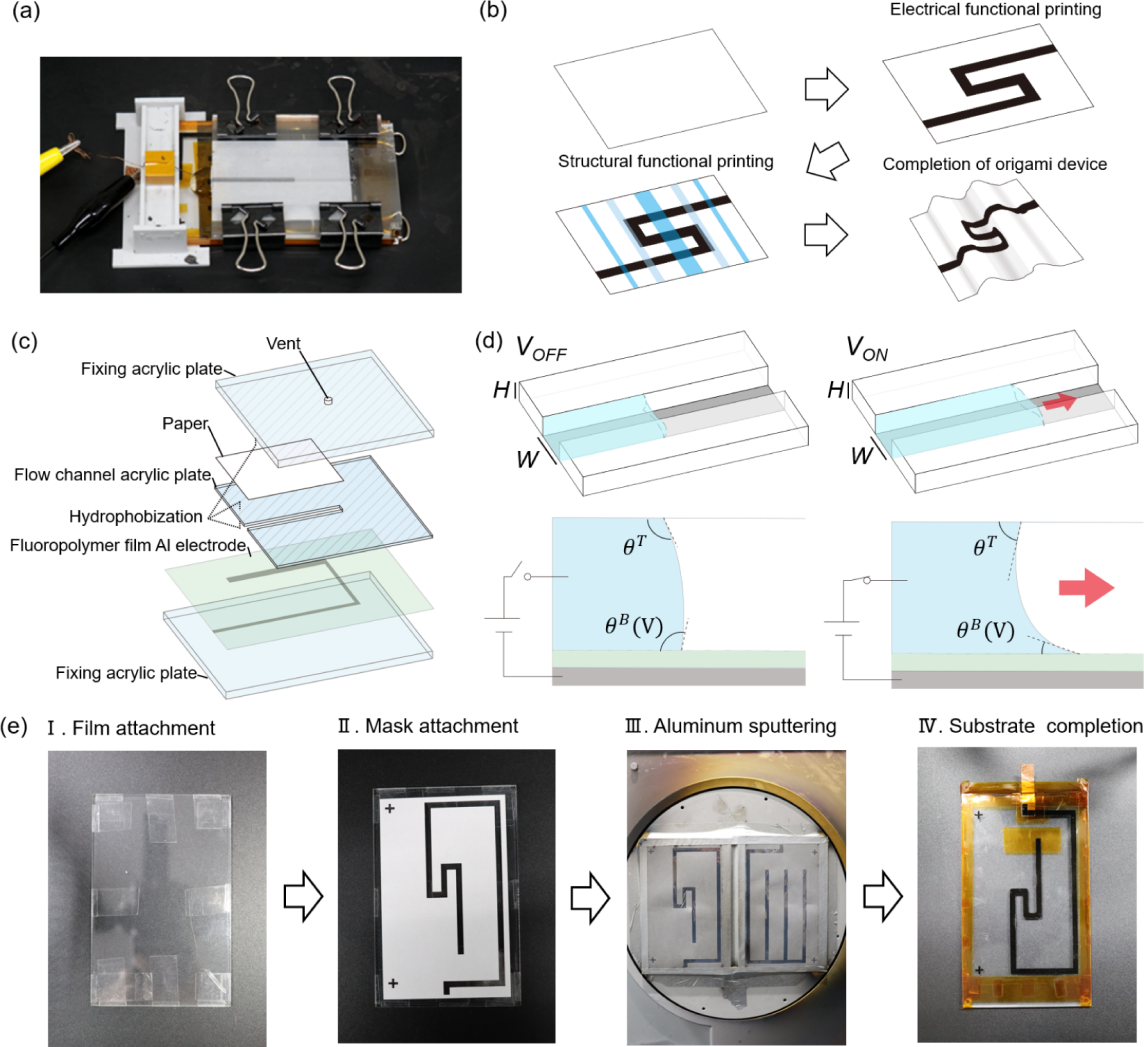
Overview and fabrication process of our proposed EWOD printing system. (a) Overview. The solution is electrically controlled in flow channels and applied onto the paper. (b) Fabrication steps of the origami device. First, the electrical function is formed by printing a conductive solution onto the paper. Then, the structure formation solution is printed onto the paper to induce self-folding for realizing the 3D origami device. (c) Printing system configuration. The solutions are applied onto the paper through the flow channels prepared in the acrylic plate. (d) EWOD operation principle. The forces are balanced, and the solution does not move before applying voltage. When voltage is applied, EWOD generates a driving force that causes the solution to flow into the channel. (e) Fabrication process of the electrode substrate. Aluminum electrodes are formed onto the fluoropolymer film by sputtering to prevent detachment.

Figure 1c shows the configuration of the printing system. First, a fluoropolymer film with sputtered aluminum electrodes is attached to a base acrylic plate, hereafter referred to as the substrate in this paper. An acrylic plate with flow channels formed by a laser cutter is placed on top of the substrate. Hydrophobic treatment was applied outside the flow channels using a water-repellent spray to prevent ink seepage. A sheet of A8-sized paper is placed on the hydrophobically treated acrylic plate, and a sealing acrylic plate is layered on top, securing the entire structure. A vent was made in the securing acrylic plate to maintain constant pressure within the flow channels. The channel structure consists of the electrode and fluoropolymer film at the bottom, acrylic side walls, and the paper as the top surface. Since the ink that enters the flow channels adheres directly to the paper, the flow channels and the printing pattern have the same shape.

Figure 1d shows the EWOD-based principle for controlling solution within the channels. The capillary force acting in a rectangular channel of width *W* and height *H* is described by Ichikawa et al. as follows.^25^

1 $$F_{\mathrm{cap}}=WH\Delta P=WH\times \gamma_{LG}\left(\frac{2\cos\theta }{W}+\frac{2\cos\theta }{H}\right)=2\gamma_{LG}\cos\theta \left(W+H\right)$$

where Δ*P* is the capillary pressure, γ_LG_ is the liquid–gas interfacial energy, and θ is the contact angle. This equation is simplified by assuming the constant contact angle. When the contact angle varies on each wall of the channel, the capillary force, which is based on the change in interfacial energy as the liquid moves within the channel, is given by Ouali et al. as follows.^26^

2 $$F_{\mathrm{cap}}=\gamma_{\mathrm{LG}}\left\{W\left(\cos\theta^{T}+\cos\theta^{B}\right)+H\left(\cos\theta^{L}+\cos\theta^{R}\right)\right\}$$

where θ with the superscripts T, B, L, and R indicates the contact angle on the top, bottom, left, and right walls, respectively. The bottom surface of the channel, which is composed of a fluoropolymer film, is hydrophobic; therefore, it causes a negative capillary force in the bottom term. As EWOD decreases the contact angle on the substrate electrode, eq 2 can be represented as follows using the Young-Lippmann equation.

3 $$F_{\mathrm{cap}}=\gamma_{\mathrm{LG}}\left\{W\left(\cos\theta^{T}+\cos\theta_{0}^{B}+\frac{1}{2\gamma_{\mathrm{LG}}}CV^{2}\right)+H\left(\cos\theta^{L}+\cos\theta^{R}\right)\right\}$$

where $\theta_{0}^{B}$ is the initial contact angle on the bottom surface of the channel before voltage application, *C* is the capacitance between the liquid and the electrode, and *V* is the applied voltage. In other words, applying the voltage enhances the capillary force, which allows control over the ingress of the solution into the flow channel.

Furthermore, capillary phenomenon is also affected by viscous resistance and gravity. The viscous resistance changes the advancing speed of the ink, and is represented as follows by Ichikawa et al.^25^

4 $$F_{\mathrm{visc}}=\frac{\pi^{4}\mu }{8\varepsilon \left\{1-\frac{2\varepsilon }{\pi }\tanh\left(\frac{\pi }{2\varepsilon }\right)\right\}}x\frac{dx}{dt}$$

where μ is the viscosity of the liquid, and ε is the aspect ratio of the channel, defined as ε = *H*/*W*. When ε < 1, 1/ε is substituted with ε. In both cases, the closer the aspect ratio is to 1, meaning the more square the flow channel becomes, the lower the viscous resistance. The influence of gravity can affect the system in two ways, namely via the flow channel inclination and hydrostatic pressure. However, in this system, the flow channel has no inclination, therefore the effect of gravity due to inclination is negligible.

Figure 1e shows the fabrication process of the electrode substrate. First, the aluminum electrodes were deposited onto a fluoropolymer film by sputtering. Directly patterning the electrode on the fluoropolymer film enhanced the adhesion between the dielectric layer and the electrode, preventing a decline in the EWOD effect. The electrode film was placed on the base acrylic plate with facing down the electrode side. Kapton tape known for its insulation properties and resistance to detachment upon exposure to solutions was used to secure the film.

### Performance Evaluation of Solution Manipulation

The controllability of the solution in the EWOD printing system was evaluated with the formation time of the printing line. Since uniform solution penetration into the paper is desirable, a shorter time to form printing line is preferable. The effects of input voltage waveform, maximum voltage, frequency, and flow channel geometry on solution controllability were examined in order to establish the design guidelines of the printer system.

As shown in Figure 2a, the solution behavior during the formation of printing line was classified into four patterns (Patterns A–D). These patterns result from the interplay among three forces: the capillary force without voltage application *F*_cap_(0), the capillary force with voltage application influenced by EWOD *F*_cap_(*V*), and the resistance to solution entry *F*_visc_. Pattern A represents the most desirable condition, where the solution does not enter the flow channel before applying voltage, and completes the printing line formation by applying voltage. This occurs when *F*_cap_(*V*) > *F*_visc_ > *F*_cap_(0). Pattern B occurs when ink enters the flow channel before applying voltage, which happens when *F*_cap_(0) > *F*_visc_. For Pattern B, the formation time of the printing line *T*_f_ is represented as 0 s on the graph. Pattern C occurs when ink does not enter the flow channel even after the voltage is applied, which happens when *F*_visc_ > *F*_cap_(*V*). For Pattern C, *T*_f_ is twice the maximum printing line formation time of Pattern A in each graph. Pattern D occurs when the ink enters the flow channel after voltage is applied, however stops midway through the flow channel. The pattern happens when *F*_visc_ ≥ *F*_cap_(*V*). For Pattern D, *T*_f_ is shown as 1.5 times the maximum printing line formation time of Pattern A in each graph.

**Figure 2:**
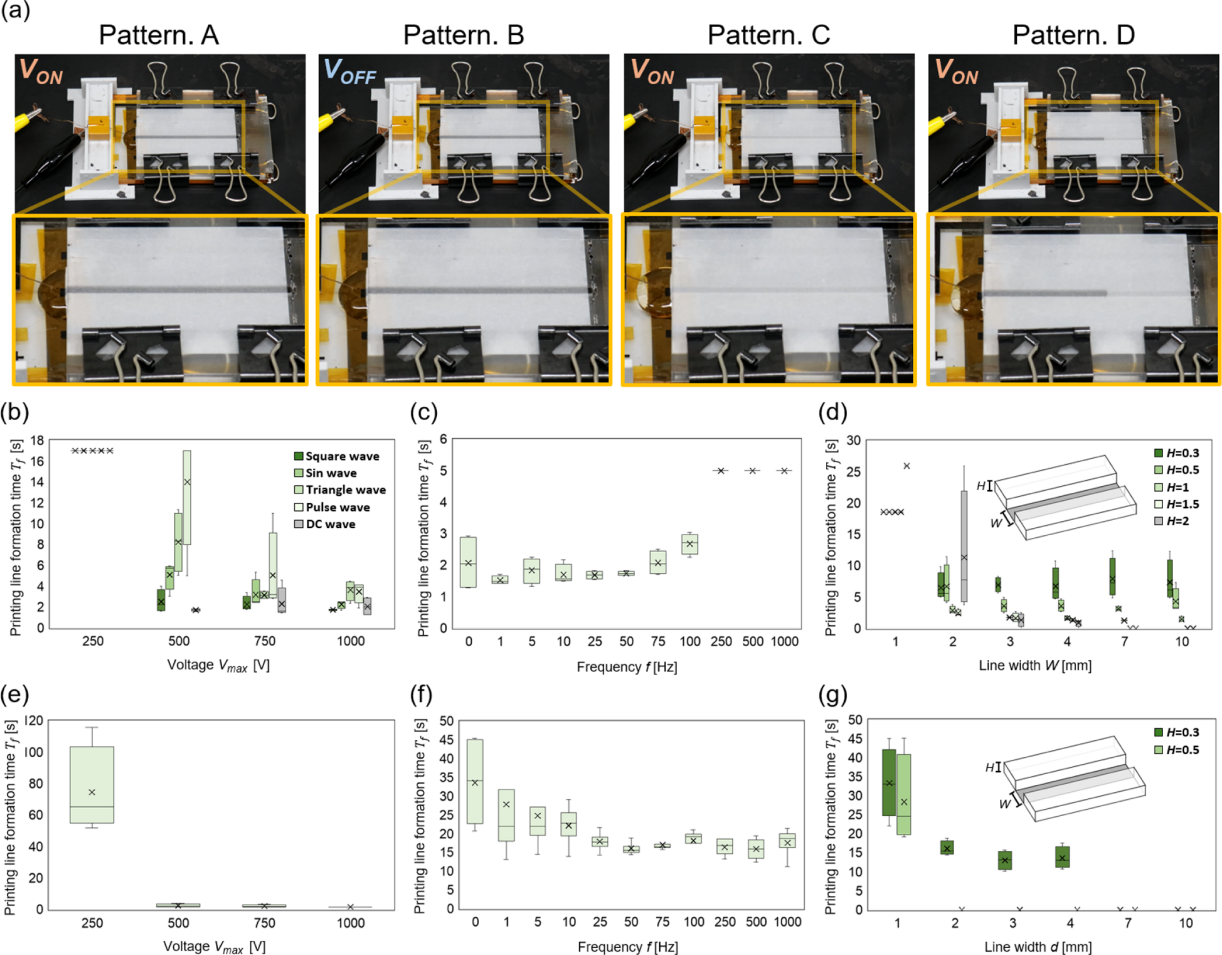
Input control signal and channel design study for printed line formation. (a) Classification for solution behavior. The solution behavior was classified into four. Pattern A (Success): The solution did not enter the channel before voltage application but entered the channel and completed the formation of the printing line after voltage application. Pattern B (Failure): The solution entered the channel even before voltage application. Pattern C (Failure): The solution did not enter the channel even after voltage application. Pattern D (Failure): The solution entered the channel after voltage application, but its advancement stopped midway, and the printing line was not completed. (b) Results of *T*_f_ when varying the input voltage and waveform with structure formation solution printing. Higher input voltages resulted in rapid solution movement, with square waveforms demonstrating the best performance. (c) Results of *T*_f_ when varying the input frequency with structure formation solution printing. No response was observed at frequencies of 250 Hz or higher. (d) Results of *T*_f_ when varying the flow channel geometry with structure formation solution printing. Higher aspect ratios of the flow channel reduced viscous resistance, which allowed faster solution movement. (e) Results of *T*_f_ when varying the input voltage with conductive solution printing. Similar to the structure formation solution, the conductive solution was driven faster as the input voltage increased. (f) Results of *T*_f_ when varying the input frequency with conductive solution printing. Due to the high viscosity of the conductive solution,*T*_f_ decreased at higher frequency ranges. (g) Results of *T*_f_ when varying the flow channel geometry with conductive solution printing. The low contact angle induces capillary force in the direction of infiltration, leading to unintended line formation before the voltage is applied.

In this system, the behavior of droplet infiltration into the microchannel is governed primarily by surface tension rather than the viscosity of the solution. The flow channel is composed of a fluoropolymer film on the bottom, acrylic plates on the sidewalls, and paper on the top surface. Differences in wettability at these interfaces directly influence the liquid behavior. As shown in Table S1, both the conductive solution and the structure formation solution exhibit relatively low contact angles with the acrylic walls (conductive solution: 32.9°, structure formation solution: 69.9°), suggesting that spontaneous penetration from the sidewalls may occur even without voltage application. In contrast, the fluoropolymer film at the bottom exhibits high water repellency, preventing liquid infiltration under natural conditions. However, by applying voltage, the EWOD effect reduces the contact angle on the bottom surface, thereby inducing liquid ingress into the channel. Given these differences in wettability across the materials, adjusting the surface area ratio between the fluoropolymer film and the acrylic walls in the cross-section of the channel may provide a means to control liquid infiltration pathways and response behavior. Such control enables the realization of the desired Pattern A operation.

Initially, the voltage waveform applied for solution control were examined by comparing their effects under various maximum voltages. The tested waveforms included square wave, sin wave, triangular wave, pulse wave, and direct current (DC), with maximum voltages of *V*_max_ = 250, 500, 750, and 1000 V, and a frequency of *f* = 50 Hz. The flow channel dimensions were *W* = 3 mm and *H* = 1 mm, and four trials were conducted for each voltage waveform. Figure 2b shows the results of *T*_f_ obtained with the structure formation solution. The plots represent the average values in the four trials, and the error bars indicate the standard deviation. Except for all waveforms at *V*_max_ = 250 V and one instance of *V*_max_ = 500 V of pulse wave, Pattern A was achieved, which led to successful formation of the printing line. As the maximum voltage increased, the probability of successful line formation rose, while *T*_f_ decreased. It was observed that with higher input energy, the driving force based on EWOD for the solution increased, and the results indicated that the system functioned as designed.

In comparison of *T*_f_ based on voltage waveforms, the square wave and direct current (DC) showed similar results, with *T*_f_ increasing progressively in the order of sin wave, triangular wave, and pulse wave. In EWOD devices, zero-crossing AC voltage has been found effective in delaying contact angle saturation by suppressing charge trapping and ion adsorption.^27,28^ However, in this experiment, the time for DC voltage was shorter compared to AC voltages such as sin and triangular waves. It is considered that during the formation of the printing line, the triple-phase contact line is continuously advancing, and this makes it less susceptible to the effects of charge trapping. To verify this hypothesis, *W* = 2 mm was employed to reduce the EWOD effect, and a comparison was made between squarer wave and DC under conditions of *f* = 50 Hz and *V*_max_ = 1 kV.

The results indicated Pattern A for the square wave and Pattern D for the DC. It is considered that under DC voltage, as the infiltration velocity in the flow channel decreases, the influence of charge trapping increases, leading to insufficient electrical driving force. It is noted that the observed trend, namely that a higher maximum voltage leads to a higher success rate of line formation and a shorter *T*_f_, is primarily based on the results obtained using square waveforms. In contrast, other voltage waveforms, such as pulse or triangular waveforms, exhibit different trends in *T*_f_ due to waveform-specific rise times and charge trapping characteristics. Therefore, the conclusions presented in this study serve as a design guideline representative of the square waveform conditions adopted in our optimized experiments and are not necessarily generalizable to all waveform types. Based on these results, we selected the square wave as the input waveform for subsequent experiments. Furthermore, the applied voltage was set to 1 kV, as it resulted in a shorter *T*_f_ without causing dielectric breakdown.

Using the selected square wave voltage with *V*_max_ = 1 kV, the influence of frequency on *T*_f_ was investigated by changing the frequency from *f* = 0 Hz to 1 kHz. Four trials were conducted for each frequency. Figure 2c shows the results of input frequency influence. The plots represent the average values of the four trials, and the error bars indicate the standard deviation. *T*_f_ decreased in the frequency range from *f* = 0 Hz to 50 Hz, and the solution movement was interrupted during the printing line formation at frequencies of *f* ≤ 250 Hz. It is considered that the solution ceased to respond to the input frequency at *f* = 250 Hz and above. Given the small variation in *T*_f_, input voltage frequencies of *f* = 25 Hz and 50 Hz were considered appropriate to employ for driving the structure formation solution.

The effect of varying the flow channel geometry on the printing line formation was investigated. The system was prepared with flow channel heights *H* = 0.3 mm, 0.5 mm, 1 mm, 1.5 m and widths *W* = 1 mm, 2 mm, 3 mm, 4 mm, 7 mm, 10 mm respectively. *T*_f_ was examined for each configuration, with four trials conducted for each flow channel shape. Figure 2d shows the results of the effect of flow channel geometry. The plots represent the average values of the four trials, and the error bars indicate the standard deviation. As *H* increased and *W* widened, *T*_f_ was reduced. This is attributed to the greater influence of hydrostatic pressure due to gravity, considering the capillary length. Additionally, the widening of *W* increased the electrode width, which in turn increased the effective area of EWOD.

The classification of controllability showed that Pattern B occurred in all results for *H* = 1.5 mm and 2 mm with *W* = 7 mm and 10 mm, and once for *H* = 2 mm with *W* = 3 mmand 4 mm. Pattern D occurred in all results for *W* = 1 mm and once for *H* = 2 mm with *W* = 2 mm. All remaining results exhibited Pattern A. The results of pattern B are attributed to the balance of capillary and hydrostatic forces. The capillary force in the driving direction, generated by the acrylic sidewalls, and the hydrostatic pressure due to gravity were greater than the capillary force in the resistance direction, which was influenced by the fluoropolymer film on the bottom surface and the paper. The results for Pattern D are considered to be due to the reduction in the effective EWOD area. As *W*became as narrow as 1 mm, the electrode width also decreased, reducing the EWOD force applied to the liquid. For *H* = 0.3 mm and 0.5 mm, the reduction *T*_f_ with increasing *W* was minimal. In flow channels with low *H* and wide *W*, an uneven solution tip was formed as shown in Figure S1. This is due to the increased viscous resistance with the low *H* and *W* exceeding the capillary length. Based on the results, *H* = 1 mm was selected as the thickness of the flow channel spacer to enable rapid formation of diverse printing line widths with the structure formation solution.

To also achieve electrical functional printing, the printing line formation using the conductive solution was evaluated. The voltage waveform was a square wave at *f* = 50 Hz, and the flow channel dimensions were *W* = 3 mm and *H* = 1 mm. Four trials were conducted for each operating voltage. Figure 2e shows the experimental results for *T*_f_ at various operating voltages using the conductive solution. The plots represent the average values of the four trials, and the error bars indicate the standard deviation. For the conductive solution, Pattern A was observed even at *V*_max_ = 250 V, where the structure formation solution had previously failed to drive. This was due to the high conductivity of the solution, which enhanced the driving force via EWOD.^29^ However, due to the high viscosity of the solution itself, *T*_f_ was prolonged. The fluoropolymer film experienced dielectric breakdown at *V*_max_ = 1.5 kV. Based on the results, the maximum voltage used in the printer system was limited to *V*_max_ = 1 kV even when driving the conductive solution.

Using the selected square wave voltage with *V*_max_ = 1 kV, the influence of frequency on *T*_f_ was investigated by changing the frequency from *f* = 0 Hz to 1 kHz. Four trials were conducted for each frequency. Figure 2f shows the results of the influence of input frequency when using the conductive solution. The plots represent the average values of the four trials, and the error bars indicate the standard deviation. Pattern A was consistently observed across all frequencies, and *T*_f_ remained low in the range from *f* = 50 Hz to 1 kHz. Due to the higher viscosity of the conductive solution, *T*_f_ was reduced at higher frequencies *f* ≥ 50 Hz. In the case of the structure formation solution, superior driving performance were observed in *f* = 20 Hz and 50 Hz. Therefore, *f* = 50 Hz was adopted as the driving frequency suitable for both solutions. This allows control of both solutions with a single voltage waveform, which simplifies control circuit design and contributes to a more compact printer system.

The effect of varying the flow channel geometry for the conductive solution was investigated. The system was prepared with flow channel heights *H* = 0.3 mm, 0.5 mm and widths *W* = 1 mm, 2 mm, 3 mm, 4 mm, 7 mm, 10 mm respectively. *T*_f_ was examined for each configuration, with four trials conducted for each flow channel shape. Figure 2g shows the results of the influence of flow channel geometry when using the conductive solution. The plots represent the average values of the four trials, and the error bars indicate the standard deviation. Pattern A was observed at *H* = 0.3 mm with *W* = 1 mm, 2 mm, 3 mm and 4 mm, and at *H* = 0.5 mm with *W* = 1 mm, while all other conditions resulted in Pattern B. This is due to the low contact angle at each channel wall as shown in Table S1. Although the conductive solution has high viscosity, the low contact angle induces capillary force in the direction of infiltration, leading to unintended line formation before the voltage is applied. Based on these findings, *H* = 0.3 mm was selected for the conductive solution, as it enables the formation of printing line widths from *W* = 1 mm to 4 mm.

In our system, the liquid inflow is governed by the interplay between EWOD-induced contact angle variation and the flow resistance determined by the channel geometry. By switching the applied voltage, selective printing behavior of specific liquids can be controlled. This selective driving mechanism, combined with tailored channel design and driving conditions for conductive and insulating liquids, provides the basis for sequential multimaterial printing.

### Structure Printing Performance

The solution was printed using the control parameters determined in the previous section to verify the structure formation function. The self-folding mechanism adopted in this study induces bending by locally impregnating specific regions of the paper substrate with the structure formation solution. Specifically, as the solution penetrates the paper along the printed patterns, asymmetric swelling of the cellulose fibers occur, generating bending stress within the paper. Unlike the case of uniform impregnation on both sides, this method exploits unidirectional and nonuniform diffusion to achieve the intended folding direction and angle. Factors that influence the self-folding behavior include the width of the printed lines, the thickness of the paper, and the permeability of the solution. These factors have been reported to affect the final folding angle and reproducibility.^17^ In this study, the manufacturing process was optimized to enable the integrated design and printing of both folding structures and conductive patterns, taking these parameters into account.

Figure 3a shows the structure printing procedure. (i) Insert the paper into the printer system. (ii) Drive the solution by applying voltage to form the printing line. (iii) Leave the paper in the system for a solution infiltration time of *T* [s]. (iv) Remove the paper from the system and leave it for 60 min to complete the self-folding. Although 90% of the folding is completed within 5 min, the paper was left for 60 min to accurately measure the folding angle. A channel height *H* = 1 mm was adopted. To maintain a consistent external environment, all procedures were conducted inside a glovebox adjusted to a room temperature of 25 °C and a relative humidity of 50 ± 5%. Figure 3b shows the definition of the folding angle. The angle was measured by connecting three points: the midpoint of the printing line as the apex and the two ends of the paper. The measured angle was then subtracted from 180° to obtain the folding angle θ. The folding angle was analyzed using the image analysis software ImageJ.

**Figure 3:**
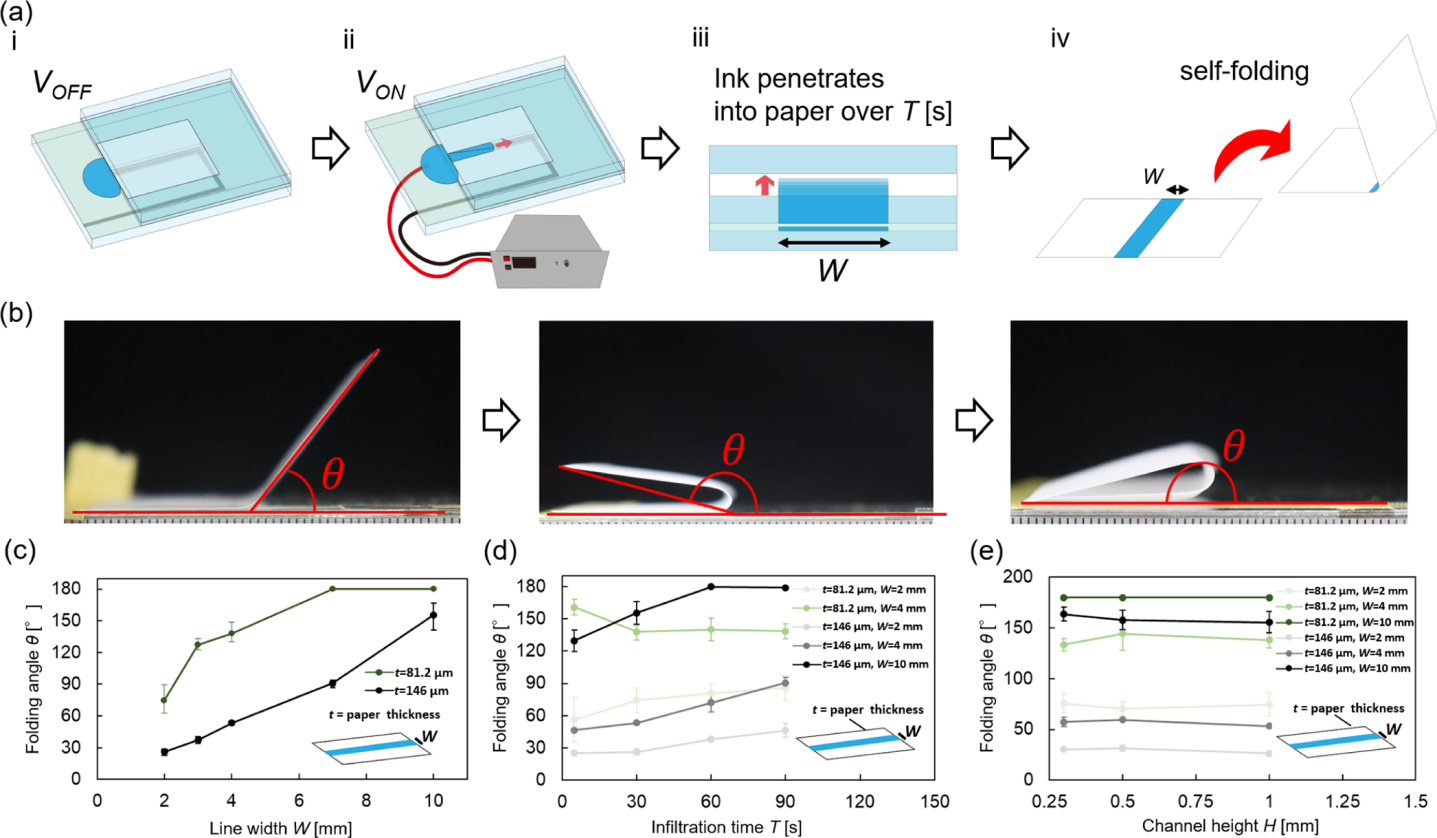
Evaluation of structure printing performance. (a) Structure printing process. The folding angle θ was investigated by controlling the printing line width *W* and the solution infiltration time *T*. (b) Definition of folding angle: The angle was measured by connecting three points—the midpoint of the printing line as the apex and the two ends of the paper. The measured angle was then subtracted from 180° to obtain the folding angle θ. (c) Relationship between printing line width *W* and folding angle θ: As in previous studies, θ showed a monotonically increasing relationship with *W*, confirming that the system was functioning correctly. (d) Relationship between infiltration time *T* and folding angle θ: Longer infiltration times *T* were effective for thicker paper due to deeper solution infiltration. (e) Relationship between flow channel height *H* and folding angle θ: Since *H* did not have a significant impact, it was concluded that *H* = 1 mm, which showed high printing performance, is optimal.

Experiments were conducted to investigate the relationship between printing line width and folding angle using two types of paper with different thicknesses, *t* = 81.2 μm and *t* =146 μm. Three trials were conducted for each condition. The solution infiltration time of *T* = 30 s was adopted. Figure 3c shows the results of the relationship between printing line width and folding angle. The plots represent the average values of the three trials, and the error bars indicate the standard deviation. For both values of *t*, the folding angle θ increased as *W* increased. Assuming a constant folding curvature, the folding angle θ is in a proportional relationship with the printing line width *W*, as shown by eq 5.

5 θ=ρW

In particular, a proportional relationship was observed for the paper with *t* = 146 μm. When *t* = 81.2 μm, a bending angle of θ = 180° was achieved at *W* = 7 mm and *W* = 10 mm. The decrease in θ for the thicker paper is attributed to the fact that, although the generated force due to infiltration remained constant because the infiltration time was identical, the rigidity of the paper increased.

To investigate the effect of infiltration time *T* on the folding angle θ, variations in θ were examined for *T* = 5, 30, 60, and 90 s. Five conditions were tested: *t* = 81.2 μm with *W* = 2 mm and 4 mm, and *t* = 146 μm with *W* = 2 mm, 4 mm, and 10 mm. Three trials were conducted for each condition. Figure 3d shows the results of infiltration time *T* and folding angle θ. The plots represent the average values of the three trials, and the error bars indicate the standard deviation. For the sample with *t* = 146 μm, θ increased with increasing *T*. This is attributed to the increase in infiltration depth, which resulted in a larger volume generating the self-folding force. The thinner the paper, the shorter the suitable infiltration time, and for *t* = 81.2 μm, *T* shorter than 30 s is deemed appropriate to maximize the folding angle. At *t* = 146 μm, the increase in folding angle with increased infiltration time was greater for larger *W*. The thicker the paper, the deeper the solution needs to infiltrate, resulting in greater bending as *T* increases. Based on the results, it was found that the folding angle can be controlled by both *W* and *T*.

Since the amount of solution in the flow channel may affect the infiltration depth and thereby influence θ, the relationship between flow channel height *H* and folding angle θ was investigated. The experimental conditions involved varying *H* = 0.3 mm, 0.5 mm and 1 mm for papers with *t* = 81.2 μm and *t* = 146 μm. Three trials were conducted for each condition. The infiltration time was set to *T* = 30 s. Figure 3e shows the results of flow channel height *H* and folding angle θ. The plots represent the average values of the three tials, and the error bars indicate the standard deviation. The maximum value was 144° at *H* = 0.5 mm, and the minimum value was 133° at *H* = 0.3 mm. The difference between the maximum and minimum values was 10.9°, which was not a significant variation. Based on these findings, it was concluded that the flow channel height does not significantly impact structure functional printing, and *H* = 1 mm, which is suitable for printing line formation, is also appropriate for structure functional printing.

### Electronics Printing Performance and Integration

To enable printing with electrical functionality, the solution was changed to a conductive solution. Based on the previous section, the flow channel height *H* was set to 0.3 mm. Figure 4a presents the printing results and an overview of the measurement process. After forming the printing line, it was left to dry for at least 12 h. Ink stains were observed along the edges of the paper at the starting and ending points of the printing line. To eliminate these stains, 3 mm from both ends of the line were cut off. The printing accuracy was evaluated by measuring the width of the printed electrode lines. As shown in Figure 4a, a straight printing line was successfully achieved. The total length of the printing line was set to 68 mm along the long edge of A8-sized paper. The line was divided into four segments at positions 17 mm, 34 mm, and 51 mm along its length. Measurements were taken at these segment points and at both ends, resulting in a total of five measurement points. The printing line widths were set to *d* = 1, 2, 3 and 4 mm. For each line width, three samples were prepared, and their widths were measured using ImageJ.

**Figure 4:**
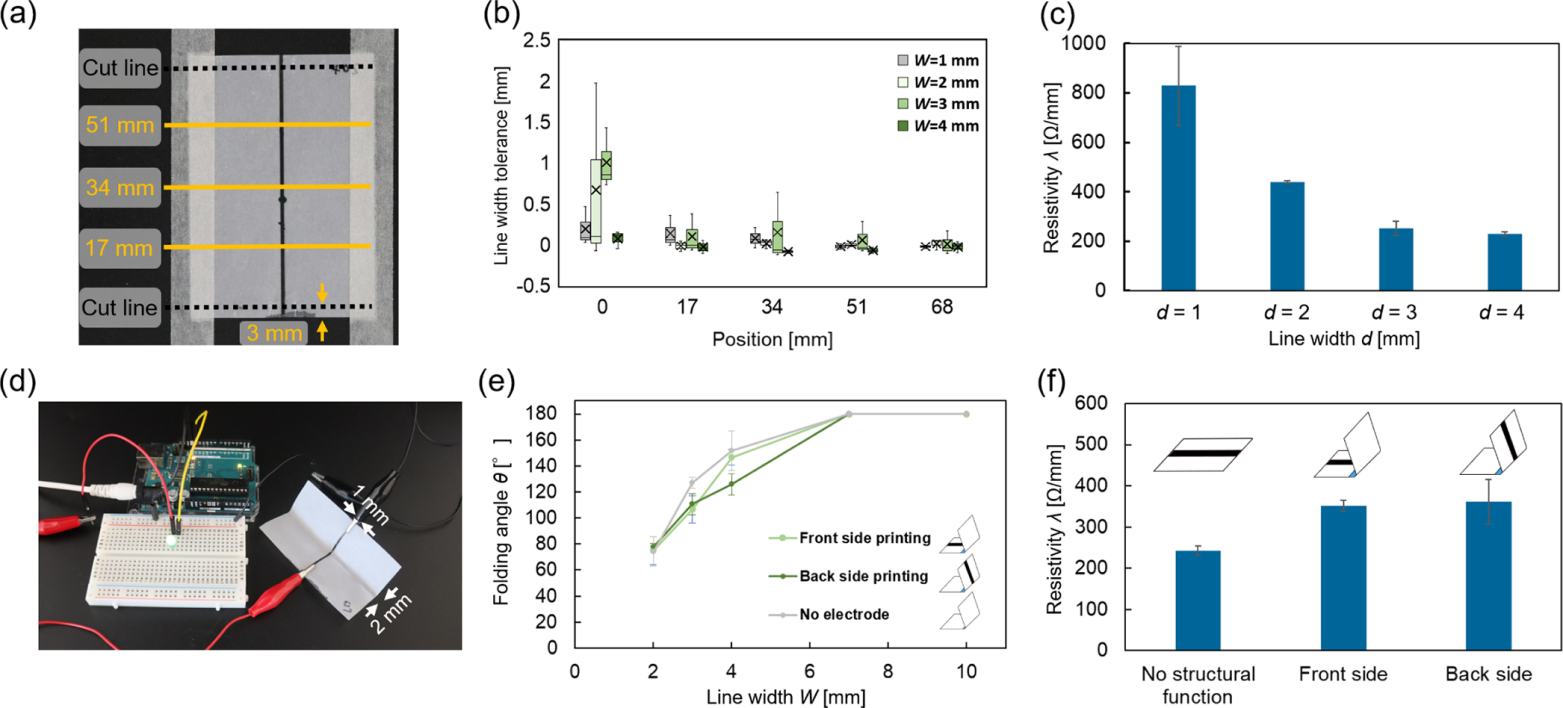
Evaluation of electrical functional printing performance and integration with structure functional printing. (a) Photograph of printed line electrode and overview of line width measurement. To mitigate ink spreading from the reservoir, 3 mm from both ends were cut off. (b) Evaluation results of electrode printing line width error. Six samples had an error of 0.1 mm or less at all five analyzed positions. (c) Linear resistivity values for *d* = 1 mm, 2 mm, 3 mm, and 4 mm. The electrode width and linear resistivity showed an inverse relationship, as expected theoretically. (d) Integration of structure functional printing and electrical functional printing. Conductivity of the folded electrode was confirmed through LED illumination. (e) Results of investigating the impact on folding angle caused by the presence of electrodes. The paper successfully underwent self-folding from either side of the electrode. (f) Results of investigating the impact of folding on electrode linear resistivity. It was demonstrated that conductivity was maintained regardless of the folding direction of the electrode.

Figure 4b shows the measurement results of the printing line width. The plots represent the average values of the three samples, and the error bars indicate the standard deviation. Near the cut line (0 mm), larger errors were observed compared to other measurement points, primarily due to ink spreading from the reservoir. In case higher accuracy is required for electrode design, it can be improved by removing more than 3 mm from the starting edge. Additionally, at observation points other than the cut line, small errors were caused by bubbles formed during the paper extraction process and misalignment between the flow channel and the paper, which resulted in irregularities in the printing. Notably, six samples exhibited errors of less than 0.1 mm at all five analyzed positions. This suggests the potential to achieve high patterning precision when printing conditions are optimized. However, this analysis was limited to specific regions of the printed area. A comprehensive evaluation across the entire pattern and further improvements in precision will be addressed as future work.

In this study, during printing with the conductive solution, ink spreading at the start and end points of the printed line, as well as slight smearing along the channel, was observed (Figure 4a,b). This phenomenon is attributed not only to the viscosity of the solution, but also to the surface tension between the liquid and the paper substrate. In particular, as shown in Table S1, the conductive solution exhibits a smaller contact angle than the structure formation solution, indicating higher wettability with paper. This makes the conductive ink more prone to lateral diffusion within the paper and localized spreading near the inlet and outlet, which can lead to variations in printing accuracy.

Additionally, nonuniformity or degradation of the liquid-repellent treatment applied to the acrylic and fluoropolymer surfaces that form the channel walls may also promote unintended spreading of the ink. These effects were quantitatively evaluated as variations in line width in Figure 4b. While the deviation tended to be larger near the inlet due to the spreading effect, it was confirmed that trimming more than 3 mm from both ends of the printed line effectively mitigated this issue. Moreover, residual liquid remaining within the spacer during paper removal may adhere to unintended areas, contributing to further smearing. To address these issues, potential countermeasures include enhancing the uniformity and durability of the liquid-repellent treatment, optimizing the formulation of the solution by considering wettability, introducing postprinting removal steps such as drying or air-blowing before separating the paper, and designing spacer structures or removal procedures that facilitate clean detachment from the paper surface.

The resistance values of the electrodes fabricated through electrical functional printing were investigated. After drying for 12 h, the resistance was measured using a multimeter (7461A, ADC) inside a humidity-controlled glovebox. The line widths were set to *d* = 1, 2, 3 and 4 mm, with three samples prepared for each width. The relationship between line width and resistance is represented by eq 6.

6 $$R=\rho_{s}\times \frac{L}{d}$$

where ρ_s_ is the sheet resistance, *d* is the electrode width, and *L* is the electrode length. Figure 4c shows the resistance values for line widths *d* = 1, 2, 3 and 4 mm. The inverse relationship between *R* and *d* indicates that the electrodes function appropriately without any nonuniformity. From eq 6, the sheet resistance was calculated to be ρ_s_ = 850 Ω/mm^2^.

Up to this point, electrical functional printing and structure functional printing have been investigated independently. Next, the simultaneous application of electrical and structure functional printing was investigated to assess their mutual interactions. Figure 4d shows an example of an origami device fabricated through the combination of electrical functional printing and structure functional printing. The paper with electrodes printed by electrical functional printing was folded using structure functional printing. The electrical and structural functional printings were applied to 81.2 μm thick paper so that their lines were orthogonal to each other. The printed line width for the electrical functional printing was set to *d* = 1 mm, while the structure functional printing width was set to *W* = 2 mm. The folding angle was θ = 86°, and it was confirmed that the electrode maintained conductivity even after folding, as demonstrated by the illumination of an LED. The paper folded inward along the printing line to form a valley fold. In Figure 4d, the electrode surface and the structure solution printing surface are on the same side, which causes the electrode section forming a valley fold. In case the electrode surface and the structure solution printing surface are on opposite sides, the electrode section forms a mountain fold. Although both the conductive solution and the structure formation solution are printed on the same paper surface in this figure, they are dispensed through independent flow channels designed with different spacer heights (conductive ink: *H* = 0.3 mm and insulating ink: *H* = 1 mm). This physical separation of the channels ensures that each solution is transported and dispensed independently, thereby avoiding structural risks of cross-contamination during material switching.

The effect of electrical functional printing on structure formation was investigated. The electrode printing line width was set to *d* = 4 mm, which is most likely to have an impact on the structure functional printing, and the resting time was set to *T* = 30 s. Three conditions were examined: without an electrode, with an electrode printed on the surface, and with an electrode printed on the backside. The folding angle was evaluated when the structure forming printing width *W* = 2 mm, 3 mm, 4 mm, 7 mm, and 10 mm. Three trials were conducted for each condition. Figure 4e shows the results of the folding angle investigation. The plots represent the average values of the three trials, and the error bars indicate the standard deviation. The paper successfully achieved self-folding even when the electrode was present, and at *W* = 7 mm and 10 mm, *θ* = 180° was achieved under all conditions. At *W* = 4 mm, the reduction rate in folding angle for cases with and without electrodes was 3.38% for surface electrodes and 17.1% for backside electrodes. When the electrode was positioned at the back, tensile stress was applied to the outer side of the fold, inhibiting folding. Based on the findings, it is demonstrated that self-folding is possible through structure functional printing, even when electrodes are formed by electrical functional printing.

Conversely, the effect of structure functional printing on electrical functionality was investigated. To examine the impact of structure formation on the electrode, the structure forming printing line width was set to *d* = 4 mm and *W* = 10 mm. Three conditions were compared: (1) only the electrode, (2) structure functional printing on the same side as the electrode, and (3) structure functional printing on the opposite side. The resistance was measured under each condition. Three trials were conducted for each condition. Figure 4f shows the results of resistance measurements when the electrode was folded. Although the resistance increased, the electrode successfully achieved self-folding without electrical breakdown. When structure functional printing was performed on the same side as the electrode, the line resistance λ = 351 Ω/mm, while it was 361 Ω/mm when performed on the opposite side, which were comparable values. Compared to the flat condition, the resistance increased by 3% when printed on the same side and by 7% when printed on the opposite side. Based on the findings, it is demonstrated that the printed electrode retains its electrical properties even when folded by printing.

### Development of a Portable Control Circuit for Miniaturization

In the previous experiments, a high-voltage amplifier (HEOPT-10B10, Matsusada Precision) and a function generator (AFG1022, Tektronix) were used as the power supply for the printer system. However, these devices are large and are not portable, which makes them unsuitable for on-demand fabrication. Therefore, a compact EWOD control circuit was developed to make the printer system portable. The control circuit was designed to output a square wave with *V*_max_ = 1 kV and *f* = 50 Hz, as determined in the experiments.

Figure 5a shows the portable multimaterial printer equipped with the developed control circuit. The printer is powered via an electrical outlet to drive the EWOD system. Figure 5b shows the developed portable circuit, while Figure 5c presents its circuit diagram. The dimensions of the fabricated circuit are as follows: length: 64.8 mm, width: 110 mm, and height: 28.0 mm. Power is supplied through an AC adapter connected to an outlet (9 V, 1 A). The input voltage is regulated to 5 V through a regulator circuit and then supplied to the Arduino, which generates control signals, and to the DC–DC converter (GP40, EMCO) for high-voltage generation. These signals feed into a full-bridge circuit to generate a zero-cross square waveform with *V*_max_ = 1 kV and *f* = 50 Hz, which is optimal for driving EWOD. The full-bridge circuit was inexpensively constructed by connecting three 450 V-rated photocouplers in series.

**Figure 5:**
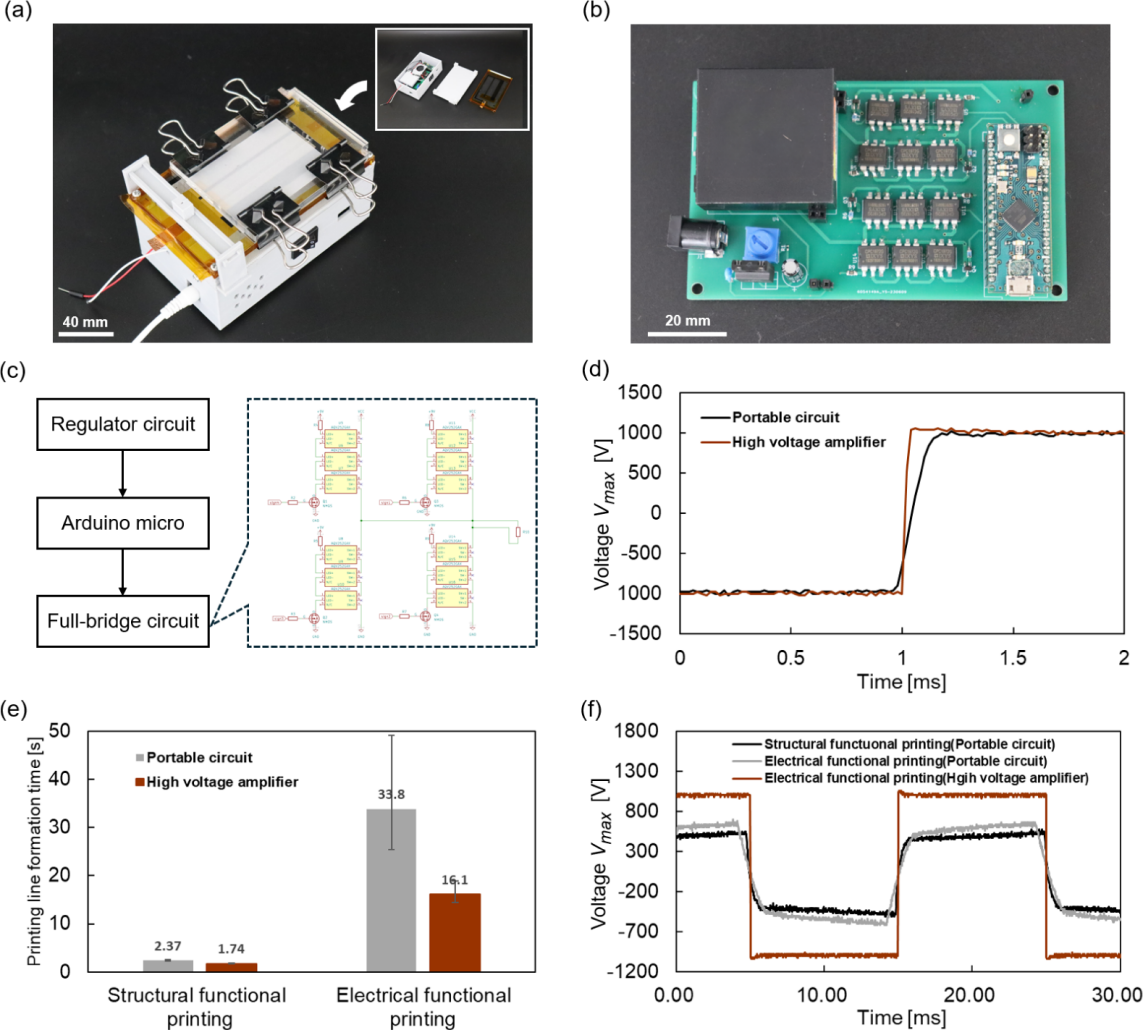
Development of a portable multimaterial printer with a compact EWOD control circuit. (a) Photograph of the portable printer system. We successfully developed a palm-sized printer system by combining the simplicity of EWOD with a developed compact control circuit. (b) Photograph of the portable control circuit. The dimensions of the fabricated circuit are as follows: length: 64.8 mm, width: 110 mm, and height: 28.0 mm. (c) Circuit configuration of the portable control circuit. A full-bridge circuit was created using phototransistors, which achieved zero-crossing voltage output to adequately control the EWOD system. (d) Comparison of the rise waveform between the portable power circuit and the high-voltage amplifier under no-load conditions. The rise time reduction was 1% of one cycle. (e) Results of *T*_f_ for the portable power supply and high-voltage amplifier. Successful printing line formation was achieved with the portable power supply. (f) Voltage waveform comparison for conductive solution printing between the portable power supply and high-voltage amplifier. The output voltage decreased due to insufficient output power from the DC–DC converter.

Figure 5d compares the rise time of the output waveform of the portable power supply and the high-voltage amplifier under no-load conditions. The rise time of the portable power supply was longer by 0.20 ms compared to the high-voltage amplifier. At 50 Hz, 0.2 ms corresponds to 1% of one cycle, which indicates sufficient output performance. In terms of size, the high-voltage amplifier has a volume of 2.8 × 10^4^ cm^3^, while the portable power supply has a reduced volume of 2.2 × 10^1^ cm^3^, which represents a size reduction of 99.9%. Figure S3 shows a size comparison between the high-voltage amplifier and the portable power supply.

To evaluate the performance of the developed printer system, structure functional printing and electrical functional printing were conducted and compared to those using a high-voltage amplifier. The paper thickness was *t* = 81.2 μm, with printing parameters set to *W* = 3 mm and *H* = 1 mm for structure functional printing, and *W* = 2 mm and *H* = 0.3 mm for electrical functional printing. Figure 5c shows the results of T_f_. Even with the portable power supply, both structure and electrical functional printing were successfully achieved in line formation. Compared to the high-voltage amplifier, *T*_f_ increased by 36.2% for structure formation printing and by 110% for electrical functional printing.

To examine differences in *T*_f_, the output voltage during printing was compared. Figure 5f shows the output voltage transition for electrical functional printing using the portable power supply and the high-voltage amplifier. The output voltage decreased by 40% with the portable power supply compared to the high-voltage amplifier. This was likely caused by the lower maximum output power of the DC–DC converter (1 W) compared to the high-voltage amplifier’s maximum output power (100 W). This is likely due to the increased capacitance between the electrode and the solution, which requires higher power. Based on these results, although T_f_ was longer with the portable power supply, successful line formation was achieved, which demonstrates that the multimaterial printer can be made portable.

### Fabrication of Origami Devices Using the EWOD Multimaterial Printer

Using the developed EWOD-based portable multimaterial printer, an origami displacement sensor and a humidity sensor were fabricated as representative examples of origami devices. Figure 6a shows a photograph of the fabricated displacement sensor, and Figure 6b shows the design printing pattern. The origami corrugated structure was formed by alternating mountain and valley folds, which resulted in a stretchable sensor with an extensibility of 100%. To form alternating mountain and valley folds, it was necessary to print the structure formation solution on both sides of the paper. Therefore, a system capable of printing on both sides simultaneously was developed.

**Figure 6:**
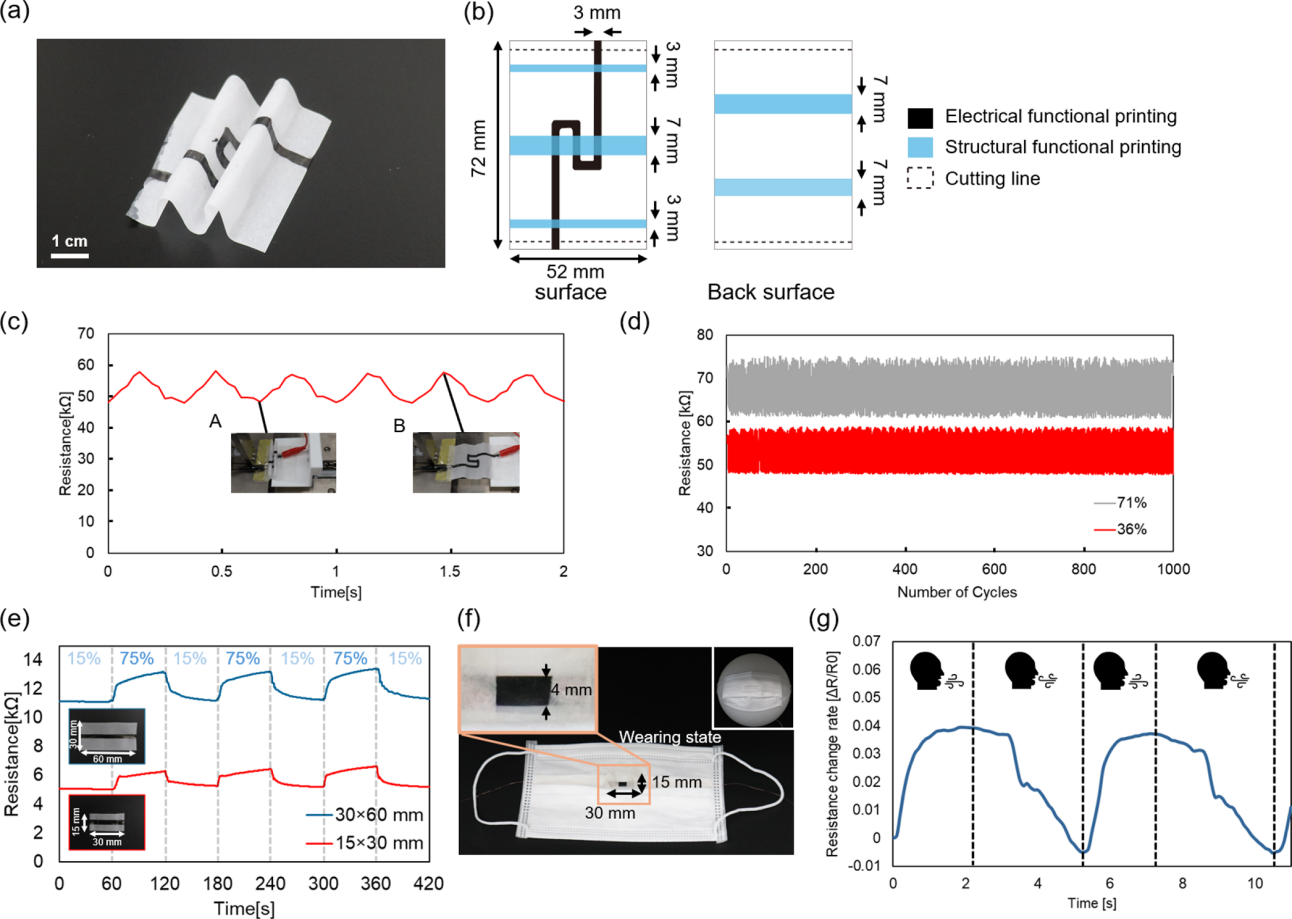
Origami devices fabricated using the EWOD-based portable multimaterial printer. (a) Stretchable origami displacement sensor. Forming an origami corrugated structure enables a stretchable sensor with 100% extension. (b) Design printing pattern of the displacement sensor. The sensor functions as a resistance-increasing displacement sensor by incorporating serpentine electrodes in the valley folds. (c) Results of repeated tensile test at 3 Hz. As designed, the displacement sensor exhibited an increase in resistance with elongation, showing a gauge factor (GF) of 0.24. (d) Results of 1000-cycle durability tests under dry (RH 36%) and humid (RH 71%) conditions. Even after 1000 stretch cycles, the resistance remained stable without drift. (e) Performance evaluation of humidity sensors fabricated using the multimaterial printer. Resistance changes in response to humidity variations were measured for sensors of different sizes. When the electrode length was doubled, the initial resistance increased by 219%. The response speed was comparable regardless of the size, indicating limited size dependence of the device. (f) Example of the breath sensor attached to the mask and worn. moisture from exhaled air and expands, resulting in an increase in the electrode resistance. The sensor does not make direct contact with the mouth and appears similar to a typical mask. (g) Breath measurement during desk work. The resistance changed by 169 Ω as it increased during exhalation and decreased during inhalation.

Figure S2 shows the double-sided simultaneous printing system. Using the *H* = 1 mm spacer, three structure formation printing lines were formed on the top surface and two lines on the bottom surface of the paper simultaneously. Subsequently, the spacer was replaced with the *H* = 0.3 mm spacer, and the serpentine electrode was printed at the center of the paper using the conductive solution. When the serpentine electrode is stretched, tensile stress is applied to the electrode. The electrode experiences tensile deformation, resulting in structural changes such as the widening of microcracks and partial rupture of the conductive network. These changes increase the overall electrical resistance. The line width was set to *d* = 3 mm, and the serpentine electrode was successfully implemented using the multimaterial printer. Videos S2 and S3 show the processes of structure functional printing and electrical functional printing.

Under a relative humidity of 36%, repeated displacements of 50 mm at a frequency of 3 Hz were applied to the sensor using a slide testing machine (EZSM3, KYOWA). The relationship between displacement and resistance was evaluated using a multimeter to assess the performance of the displacement sensor. Figure 6c shows the results of the tensile test. As designed, the displacement sensor exhibited an increase in resistance with increasing displacement, indicating successful fabrication. For a displacement of 50 mm, the resistance increased by 9.9 kΩ, and given the initial resistance of 49.1 kΩ, the gauge factor (GF) was calculated to be 0.24. Equation 7 shows the formula used to calculate the GF.

7 $$\mathrm{GF}=\frac{\Delta R/R_{0}}{\Delta L/L_{0}}$$

where Δ*R* is the change in resistance, *R*_0_ is the initial resistance, Δ*L* is the displacement, and *L*_0_ is the initial length.

Next, a durability evaluation, repeated tensile tests were conducted under both low (36% RH) and high (71% RH) humidity conditions, applying a displacement of 50 mm at a frequency of 3 Hz for 1000 cycles. Figure 6d shows the results of the durability evaluation. The device exhibited stable resistance changes throughout all 1000 cycles. Under 36% RH, in the first cycle, the resistance before stretching was 49.1 kΩ and increased to 56.9 kΩ after stretching, corresponding to Δ*R*/*R*_0_ = 15.9% change. In the 1000th cycle, the resistance before stretching was 48.2 kΩ and 56.9 kΩ after stretching, resulting in Δ*R*/*R*_0_ = 18.0% change. Under 71% RH, in the first cycle, the resistance increased from 64.8 kΩ to 72.2 kΩ (Δ*R*/*R*_0_ = 11.4% change), and in the 1000th cycle, from 60.9 kΩ to 72.0 kΩ (Δ*R*/*R*_0_ = 18.2% change).

Although slight variations were observed under both conditions, the response trend of the sensor remained consistent, and no significant performance degradation due to drift or structural failure was detected. These results confirm that the sensor exhibits stable responses and possesses excellent structural and electrical durability across a wide range of humidity environments. Additionally, it is generally accepted that acquiring a signal at 3 Hz enables the sensor to be used for measuring human motion.^30^ Furthermore, the fact that the printed electrode maintained conductivity even after 1000 cycles of deformation indicates that the conductive layer on the paper substrate possesses sufficient adhesion stability for practical use. In this study, we utilized the inherent water absorbency and porous fibrous network of paper, which allows the conductive ink to penetrate into the substrate and become physically anchored between the fibers. As a result, the conductive layer is securely fixed without requiring special surface treatments or adhesive layers, thereby enabling the structure to withstand repeated deformation without delamination. Such a functional role of paper materials has also been reported by Hu et al.,^31^ demonstrating that leveraging the structural and functional properties of paper substrates is effective for designing printed electronic devices.

Next, a humidity sensor was fabricated using the proposed printer system, and its performance was evaluated. Specifically, sensors were prepared by printing electrodes (*d* = 4 mm in width and either 30 mm or 60 mm in length) on two types of paper substrates with different dimensions (15 mm × 30 mm and 30 mm × 60 mm). Each sensor was alternately exposed to environments with relative humidity of 15% and 75% for 1 min, and the resulting resistance changes were measured. The measurement results are shown in Figure 6e. When the electrode length was doubled from 30 mm to 60 mm, the initial resistance increased by 219%. However, the response speed to humidity changes showed no significant difference, indicating that the sensor response was only minimally affected by the device size. This humidity sensor operates based on the principle that the paper substrate absorbs moisture from the ambient air, leading to swelling of its fibrous structure. This swelling causes changes in the spacing and contact conditions of the conductive paths formed within or on the surface of the paper, resulting in an increase in electrical resistance.

The resistance change during desk work was measured to evaluate the performance of the fabricated breath sensor. Figure 6f shows the sensor and how it appears when attached to a mask. The electrodes were wired with enamel-coated wires and secured with clips. The sensor did not touch the mouth when worn, and its appearance was indistinguishable from a regular mask. Figure 6g shows the rate of resistance change during desk work as measured by the breath sensor. Since the electrode resistance increases under high-humidity conditions, the resistance increases during exhalation and decreases during inhalation. During the first breathing cycle, the resistance measured 4.10 kΩ during exhalation and 4.01 kΩ during inhalation. Since the normal breathing rate at rest is 12 to 20 times per minute, the observation of two cycles of the waveform in 10 s indicates that the sensor is capable of measuring breathing. The rate of resistance change during exhalation and inhalation was Δ*R*/*R*_0_ = 0.04, and the sensor output remained stable in four measurements.

## Conclusions

In this study, we proposed a portable multimaterial printer system using electrowetting on dielectric (EWOD) technology, which allows on-demand fabrication of origami devices. Using EWOD technology, capable of printing both insulating and conductive solution, we successfully printed both structure and electrical functions on the paper substrates. Furthermore, the developed printer system allowed for the successful fabrication of origami devices. By adjusting the flow channel geometry, we identified optimal conditions for each printing method, which enabled the printing of electrical patterns. Experimental results confirmed that a flow channel height of 1 mm was optimal for structure functional printing, while 0.3 mm was optimal for electrical functional printing. As applications, we fabricated a displacement sensor and a breath sensor, which demonstrates that even when integrating electrical and structure functionalities, device fabrication is possible without compromising either function. The displacement sensor showed high durability and was able to withstand 1000 cycles in a sliding test. Eventually, a compact power circuit was developed, successfully making the printer portable.

In the future, improvements in materials, design, and control methods will aim to increase the complexity and accuracy of printed lines. On-demand fabrication of origami devices could contribute to smart packaging adapted to fruit size and shape in agriculture, or disposable wearable devices tailored to patient body shapes in the medical field. The key feature of this study lies in the combination of EWOD control and flow channel structure design, which enables selective control and printing of different liquids under optimized conditions. This approach not only allows the system to function as a portable multimaterial printer but also offers potential for broader fluidic applications such as reagent handling, reaction control, and sensing in lab-on-chip. Since the concept of electrically driving solution in a microchannel using EWOD has not yet been proposed, this study also holds promise for applications in microfluidics and lab-on-chip fields where EWOD technology is anticipated to make significant contributions.

## Experimental Section

### Materials

The conductive ink used in this study was a commercially available carbon-based ink (11806, BokuUndo Co., Ltd., Japan). The structure formation solution was prepared according to the formulation reported in our previous work.^10^ Substrate materials included A8-sized paper (thickness: 81.2 or 128 μm), fluoropolymer film with sputtered aluminum electrodes, and laser-cut acrylic plates for channel formation. Hydrophobic treatment of the acrylic surface was performed using a water-repellent spray (FK31850371, FK Co., Ltd., Japan).

### EWOD Printer Fabrication

The EWOD-based multimaterial printer was constructed by layering the following components: a base acrylic plate with attached fluoropolymer film bearing aluminum electrodes (sputtered), a laser-cut acrylic plate forming the flow channels, and an upper sealing acrylic plate. The paper was placed between the acrylic plates, forming the top surface of the flow channels. A vent was added to the sealing plate to maintain internal pressure equilibrium. The channel heights were determined by spacer thicknesses (conductive solution: 0.3 mm, structure formation solution: 1.0 mm).

### Printing Process

To initiate the printing process, the solution was dispensed onto the inlet of the flow channel. Upon voltage application, the ink was drawn into the channel and printed onto the paper. For structure printing, the self-folding behavior was induced by asymmetric infiltration of the structure formation solution. The infiltration time *T* was controlled within a range of 10 to 90 s before drying. Folding angles were analyzed using ImageJ. For conductive ink printing, a drying period of at least 12 h was applied before evaluating pattern accuracy and resistance.

### Double-Sided Printing

To achieve simultaneous printing on both sides of the paper, a new system was developed. Structure formation lines were printed on both the top and bottom surfaces using separate flow channels, enabling the creation of complex folding structures, such as alternating mountain and valley folds.

### Electrical Resistance Measurement

After drying, the resistance of the printed electrodes was measured using a digital multimeter (7461A, ADC) inside a humidity-controlled glovebox. The sheet resistance was calculated from the resistance, width, and length of the printed lines. The resistance of the serpentine electrodes was also measured to evaluate their electrical performance.

### Durability and Sensor Evaluation

The printed displacement sensor was evaluated using a sliding machine (EZSM3, KYOWA) under 3 Hz cycling at a displacement of 50 mm. The gauge factor was calculated from the change in resistance as a function of displacement. The breath and humidity sensors were evaluated by measuring the variation in resistance in response to environmental humidity (15–75% RH) and exhalation, respectively. Resistance was recorded using enamel-coated wires and clips while the sensor was worn on a mask.

### Control Circuit Design

The portable control circuit (64.8 mm × 110 mm × 28.0 mm) consisted of an Arduino Nano, a 5 V regulator, and a DC-DC converter (GP40, EMCO) generating high voltage. A full-bridge configuration using three 450 V-rated photocouplers enabled the generation of a zero-crossing square wave output.

## Supplementary Material



## Notes

Materials and methods (pdf), rinting process using the EWOD printing system (Video S1), serpentine electrode printing by electrical function printing (Video S2), printing structure of origami stain sensor using double-side structure formation printing (Video S3) (ZIP)
