# Supplementary material for: Electrowetting on Dielectric (EWOD) Based Portable Multimaterial Printer To Fabricate Origami Devices
Source: ACS Appl Mater Interfaces. 2025 Jul 31;17(32):46442–55. doi: 10.1021/acsami.5c12629 (PMC12356536; doi:10.1021/acsami.5c12629)
Supplement: Supplementary file 1 [file am5c12629_si_001.zip › supporting information/supporting information.docx]

**Supporting Information**

**Electrowetting on dielectric (EWOD) based portable multi-material printer to fabricate origami devices**

Yuhi Watanabe,^†^ Atsushi Matsushita,^†^ Mutsuki Matsumoto,^†^
Yusuke Akitsu,^†^ Yu Kuwajima,^⁋^ and Hiroki Shigemune*^,‡^

†Electrical Engineering and Computer Science, Graduate School of Engineering and Science, Shibaura Institute of Technology, 3-7-5, Toyosu, Koto-ku, Tokyo, 135-8548, Japan

⁋ Department of Mechanics, Mathematics and Management (DMMM), Politecnico di Bari,
Via Orabona 4, 70125, Bari, Italy.

‡College of Engineering, Shibaura Institute of Technology,
3-7-5, Toyosu, Koto-ku, Tokyo, 135-8548, Japan

Email: hshige@shibaura-it.ac.jp

**Structural formation printing**

Figure S1 shows the printed line formed with an uneven liquid tip ($H=0.3 \mathrm{mm}, W=7 \mathrm{mm}$). According to Equation (4), the viscous resistance of the solution increases as the aspect ratio of the flow channel shape increases. Therefore, at $H=0.3 \mathrm{mm}$ and $W=7 \mathrm{mm}$, the viscous resistance increased, and because the width $W$ was more than twice the capillary length, the printed line formed unevenly.


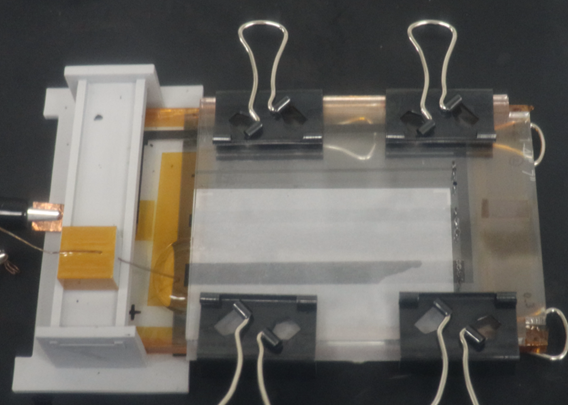


Figure S1. Printed line formed with an uneven liquid tip ($H=0.3 \mathrm{mm}, W=7 \mathrm{mm}$).

**Double-side printing system**

Figure S2 shows the system configuration for double-side printing. The system from Figure 1(c) was modified by adding an acrylic flow channel plate and an electrode film to the top of the system. The upper acrylic flow channel plate has an inlet located at a higher position, and the electrode film was attached to the side of the fixed acrylic plate to enhance the EWOD effect.


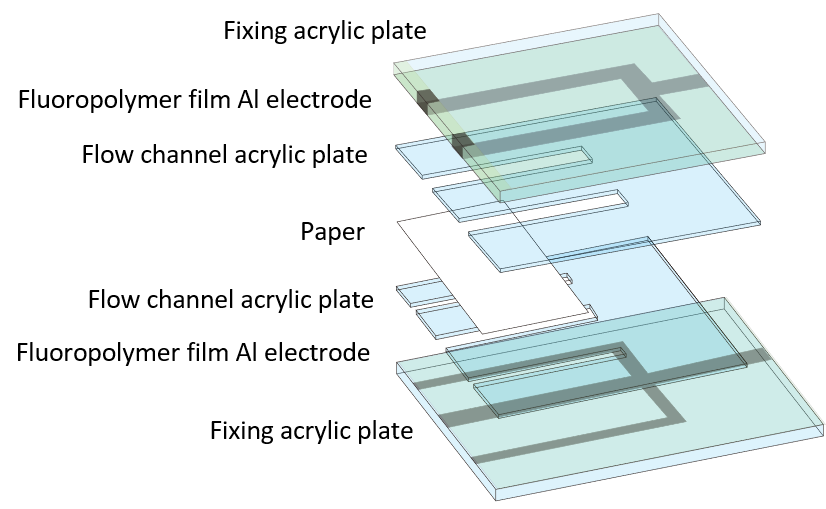


Figure S2. Configuration of double-side printing system.

**Size comparison between high-voltage amplifier and developed portable power supply**

Figure S3 shows the size comparison between the high-voltage amplifier and the developed portable power supply. The high-voltage amplifier has a volume of $2.8\times{10}^{4} \mathrm{cm}^{3}$, while the portable power supply has a volume of $2.2\times{10}^{1} \mathrm{cm}^{3}$, resulting in a 99.9% reduction in size.

5 cm


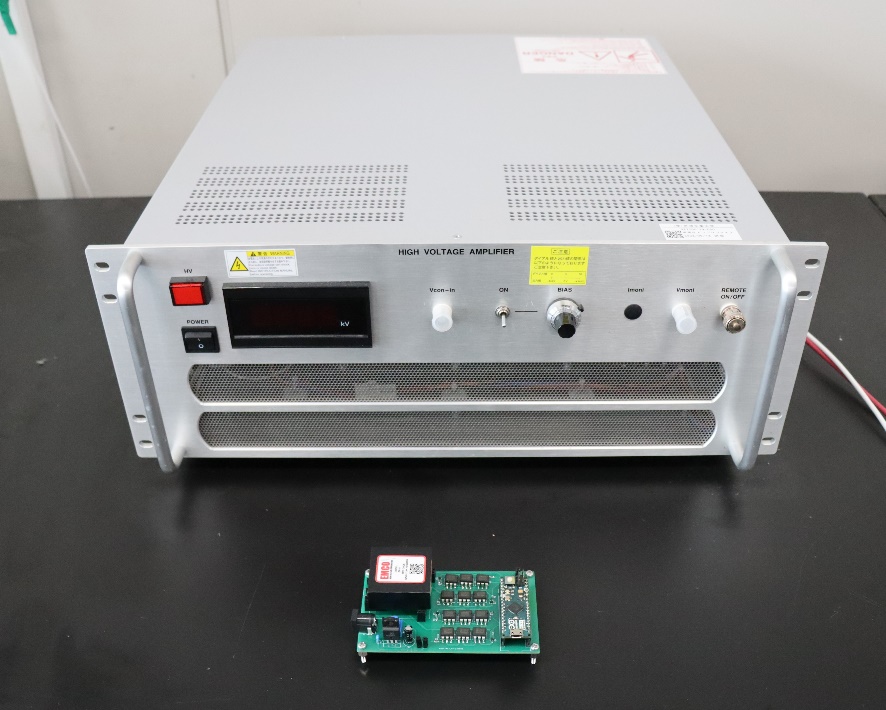


Portable circuit

High voltage amplifier

Figure S3. Size comparison between high-voltage amplifier and our developed portable power supply.

**Contact angles of structural formation solution and conductive solution with various substrates**

Table S1 shows the contact angles of the structural formation solution and the conductive solution with various substrates. The smaller contact angle between the solution and the acrylic flow channel plate facilitates entry of the solution to the flow channel when voltage is applied. When the contact angle is too small, droplets can enter the channel without applying the voltage, making it necessary to appropriately control the viscous resistance through the design of the channel aspect ratio. Compared to the structure formation solution, the conductive solution has a smaller contact angle, allowing it to infiltrate the flow channel more easily, thereby preventing Patterns C and D from occurring.

Table S1. Contact angles of structure formation solution and conductive solution with various substrates.

|  | Fluoropolymer film | Paper | Acrylic plate |
| --- | --- | --- | --- |
| Contact angles with  structure formation solution  (degree) | 111 | 103 | 69.9 |
| Contact angles with  conductive solution  (degree) | 76.6 | 38.4 | 32.9 |

**Supplementary video description**

**Video S1. Printing process using the EWOD printing system.**

The solution is dropped onto the system while the paper is held in place, and the voltage applied between the solution and the electrode causes the solution to form the printing pattern. The flow channel shape was $H=1 \mathrm{mm}, W=3 \mathrm{mm}$, and the applied voltage was a square waveform with $f=50 \mathrm{Hz}$ and $V_{max}=1 \mathrm{kV}$.

**Video S2. Serpentine electrode printing by electrical functional printing.**

The EWOD multi-material printer enables the printing of serpentine electrodes. Printing serpentine electrodes in the valley folds resulted in the fabrication of a resistance-increasing strain sensor.

**Video S3. Printing structure of origami stain sensor using double-side structure formation printing.**

Using the double-side printing system shown in S2, three structure formation lines were printed on the same side as the serpentine electrodes, and two structure formation lines were printed on the opposite side. The printing line formation starts from the bottom side because of the influence of hydrostatic pressure due to gravity.
